# Supplementary material for: CaMKK2 in myeloid cells is a key regulator of the immune-suppressive microenvironment in breast cancer
Source: Nat Commun. 2019 Jun 4;10:2450. doi: 10.1038/s41467-019-10424-5 (PMC6547743; doi:10.1038/s41467-019-10424-5)
Supplement: Supplementary file 1 — Supplementary information [file 41467_2019_10424_MOESM1_ESM.pdf]

Supplementary Information

CaMKK2 in myeloid cells is a key driver of the immune-suppressive microenvironment in breast cancer

Luigi Racioppi et al.

This PDF file includes:

Supplementary Tables 1 to 3

Supplementary Figures 1 to 12

Supplementary Data Files 1 to 2 (provided as .xlsx files)

Supplementary Table 1. CaMKK2 expression by tumor grade. Immunohistochemical analysis of CaMKK2 expression in human breast cancer tissue microarrays from two independent datasets (Vienna and Roswell Park Cancer Institute, RPCI). CaMKK2 expression was determined to be low (<2) or high (>=2) and correlated with tumor grade (1, 2 and 3). A Fisher's exact test was used to determine p-values for the likelihood of association. N=sample numbers, *ns*=not significant (p> 0.05).

	**Vienna**				**RPCI**				**Combined**			
**CaMKK2**	N	1	2	3	N	1	2	3	N	1	2	3
**Low**	32	22%	63%	15%	54	2%	13%	85%	86	9%	31%	60%
**High**	15	7%	53%	40%	14	7%	7%	86%	29	7%	31%	62%
p-value	*Ns*				*ns*				*ns*			

Supplementary Table 2. Antibodies and reagents used for flow cytometry.

Specificity (myeloid panel)	Clone	Fluorochrome	Company	Catalog #	Dilution*
anti-mouse CD24	M1/69	BV711	BD Biosciences	563450	1:2000
anti-mouse CD24	M1/69	FITC	BioLegend	101805	1:400
anti-mouse Ly6C	HK1.4	PerCP-Cy5.5	BioLegend	128012	1:200
anti-mouse/human CD11b	M1/70	PE	BioLegend	101207	1:200
anti-mouse F4/80	BM8	PE-Cy7	BioLegend	123114	1:400
anti-mouse CD64	X54-5/7.1	APC	BioLegend	139306	1:200
anti-mouse Ly6G	1A8	AF-700	BD Biosciences	561236	1:200
anti-mouse IA/IE (MHC II)	M5/114.15.2	APC/Cy7	BioLegend	107628	1:400
anti-mouse CD45	30-F11	Pacific Blue	BioLegend	103126	1:200
anti-mouse CD45	30-F11	BV510	BioLegend	103138	1:200
anti-mouse CD206	C068C2	BV650	BioLegend	141723	1:200
anti-mouse CD206	C068C2	APC	BioLegend	141708	1:200
anti-mouse CD11c	N418	BV711	BioLegend	117311	1:200
**Specificity (lymphoid panel)**	**Clone**	**Fluorochrome**	**Company**	**Catalog #**	**Dilution**
anti-mouse CD4	GK 1.5	FITC	Thermo Fisher Scientific	11-0041-82	1:200
anti-mouse CD4	RM4-5	PerCP-Cy5.5	Invitrogen	45-0042-82	1:200
anti-mouse CD3	17A2	PE	BioLegend	100206	1:200
anti-mouse CD3	145-2C11	PerCP-Cy5.5	BioLegend	100328	1:200
anti-mouse 41-BB	17B5	PE/Cy7	Invitrogen	25-1371-80	1:200
anti-mouse CD279 (PD-1)	RMPI-30	APC	Thermo Fisher Scientific	17-9981-82	1:200
anti-mouse CD8a	53-6.7	AF700	Thermo Fisher Scientific	56-0081-82	1:200
anti-mouse CD8a	53-6.7	AF700	Thermo Fisher Scientific	56-0081-82	1:200

anti-mouse CD8a	53-6.7	APC	BioLegend	100712	1:200
anti-mouse CD69	H1.2F3	APC/Cy7	BioLegend	104526	1:200
anti-mouse CD45	30-F11	Pacific Blue	BioLegend	103126	1:200
anti-mouse CD45.2	104	APC/Cy7	Thermo Fisher Scientific	560694	1:200
anti-mouse CD223 (LAG-3)	C9B7W	BV650	BioLegend	125227	1:200
anti-mouse CD366 (TIM-3)	RMT3-23	BV711	BioLegend	119727	1:200
anti-mouse NK1.1	PK136	PE	Thermo Fisher Scientific	12-5941-82	1:200

**Specificity (BMDM and T cells panel)**	**Clone**	**Fluorochrome**	**Company**	**Catalog #**	**Dilution**
anti-mouse/human CD11b	M1/70	PE-Cy7	BioLegend	101216	1:200
anti-mouse CD40	3/23	PE-Cy5	BioLegend	124618	1:200
anti-mouse CD86	GL-1	PE-Cy5/APC	BioLegend	105016	1:200
anti-mouse CD80	16-10A1	FITC	BioLegend	104706	1:200
anti-mouse CD11c	N418	FITC	BioLegend	117306	1:200
anti-mouse CD11c	N418	PE	BioLegend	117308	1:200
anti-mouse CD11c	N418	APC	BioLegend	117310	1:200
anti-mouse F4/80	BM8	APC	BioLegend	123116	1:200
anti-mouse F4/80	BM8	PE-Cy7	BioLegend	123114	1:400
anti-mouse IA/IE (MHC II)	M5/114.15.2	APC/Cy7	BioLegend	107628	1:400
anti-mouse H-2 (MHC I)	M1/42	FITC	BioLegend	125508	1:200
anti-mouse CD3	17A2	PE	BioLegend	100206	1:200
anti-mouse CD8a	53-6.7	PE-Cy7	BioLegend	100722	1:200
anti-mouse CD4	GK1.5	APC/Cy7	BioLegend	100414	1:200

Cell viability and trackers reagents			Company	Catalog #	
Fixable Viability Dye eFluor® 450			Thermo Fisher Scientific	65-0863-14	
CellTrace™ CFSE Cell Proliferation Kit			Invitrogen	C34554	

*All antibodies were diluted in PBS-2% FCS, 2mM EDTA; and used at 100 μL/1000000 cells.

Supplementary Table 3: Primers used for real-time PCR and genotyping

qPCR Primers	Sequence (5'-3')
Gapdh-F	CCTGGAGAAACCTGCCAAGTATG
Gapdh-R	AGAGTGGGAGTTGCTGTTGAAGTC
Arg1-F	GCAAGGTGATGGAAGAGAC
Arg1-R	CATCGACATCAAAGCTCAGG
Nos2-F	GCAAACATCACATTCAGATCCC
Nos2-R	TCAGCCTCATGGTAAACACG
Camkk2-F	CATGAATGGACGCTGC
Camkk2-R	TGACAACGCCATAGGAGCC
Retnla-F	CAAGGAACTTCTTGCCAATCCAG
Retnla-R	CCAAGATCCACAGGCAAAGCCA
Chi3l3-F	TACTCACTTCCACAGGAGCAGG
Chi3l3-R	CTCCAGTGTAGCCATCCTTAGG
Cxcl9-F	GGAGTTCGAGGAACCCTAGTG
Cxcl9-R	GGGATTTGTAGTGGATCGTGC
Cxcl10-F	CCAAGTGCTGCCGTCATTTTC
Cxcl10-R	GGCTCGCAGGGATGATTTCAA
Cxcl11-F	CGCCCCTGTTTGAACATAAG
Cxcl11-F	CTGCTGAGATGAACAGGAAGG
Cxcl14-F	CCACTCTCGACCCTACATGG
Cxcl14-R	GGCCCCCAAAGTGACATTTATT
Ccl5-F	CCACTTCTTCTCTGGGTTGG
Ccl5-R	TGCCCACGTCAAGGAGTAT
Gzmb-F	CCACTCTCGACCCTACATGG
Gzmb-R	GGCCCCCAAAGTGACATTTATT
Prf1-F	AGCACAAGTTCGTGCCAGG
Prf1-R	GCGTCTCTCATTAGGGAGTTTTT
Foxp3-F	CCCATCCCCAGGAGTCTTG
Foxp3-R	ACCATGACTAGGGGCACTGTA
Pdcd1-F	ACCCTGGTCATTCACTTGGG
Pdcd1-R	CATTTGCTCCCTCTGACACTG
Genotype Primers	Sequence (5'-3')
Camkk2$^{-/-}$ F	CGTATAATGTATGCTATACGAAG
Camkk2$^{-/-}$ R	TTGAACTCCTGACCTTCGG
Camkk2$^{+/+}$ R	CGTCTTTCTTTTTTTGGGGGT
Camkk2$^{+/+}$ R	CCTTGTTTGGGGAATGTGGAATAG
LysM-Cre F	ACCAGGTTCGTTCACTCATGG
LysM-Cre R	AGGCTAAGTGCCTTCTCTACA
Camkk2$^{fl/fl}$ F	CATTACCACTGCTCTTGGGTTCC
Camkk2$^{fl/fl}$ R	GGGGAGATAACTGCCTTTGGAG

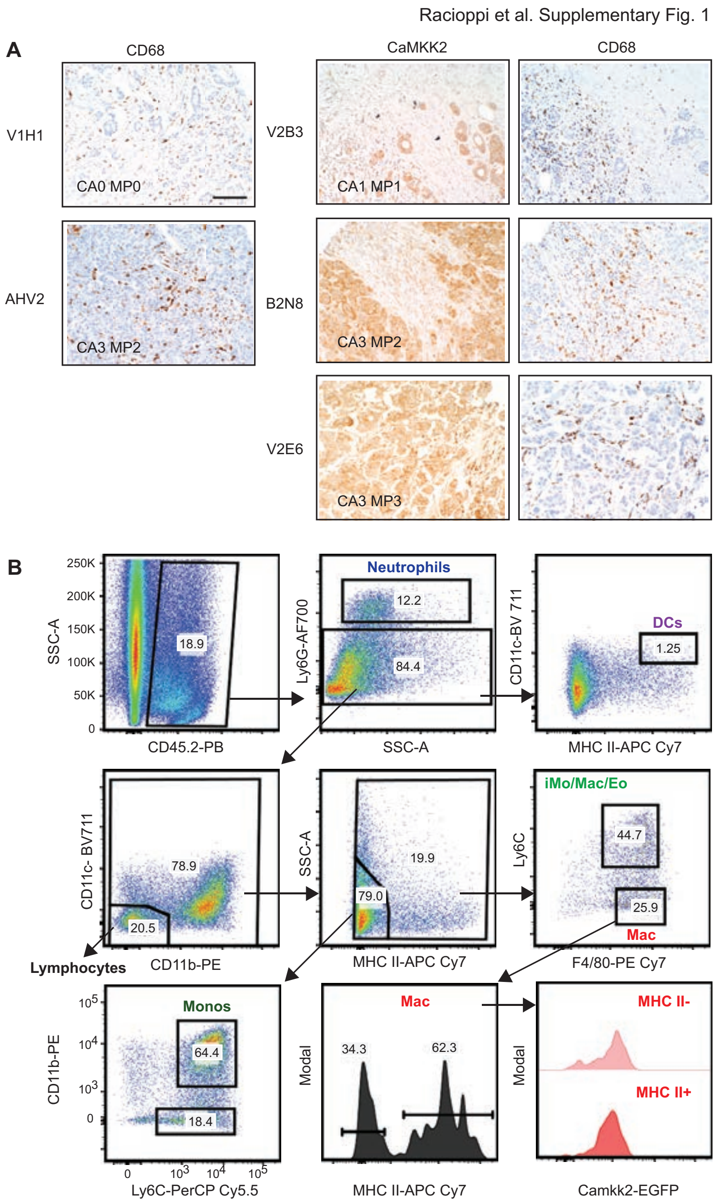

Supplementary Figure 1. CaMKK2 is expressed in mammary tumors. (A) Human breast cancer sequential sections were stained with anti-CaMKK2 or anti-CD68 antibodies. The expression of CaMKK2 in cancer cells (CA) and stromal cells/macrophages (MP) were assigned a score by a board-certified pathologist according to the staining intensity (1=low, 3=high). Sample identification numbers have been reported on the left side of each group of sections. The staining for CaMKK2 of V1H1 and AHV2 are shown in Fig. 1A (left and right, respectively). Scale bar = 100 µm. (B) E0771 cells (4 x 10^5 cells/mouse) were inoculated into the mammary fat pad of *Camkk2*-EGFP Tg mice. Subsequently, tumors were removed, digested with collagenase and DNase I and single cell suspensions were stained for immune cell markers. Tumor-infiltrating lymphocytes (including B and T cells), neutrophils, and MHC II^- monocytes (Monos) were identified according to the reported gating strategy. Gates including cell populations enriched for dendritic cells (DCs), macrophages (Mac), MHC II+ monocytes/Ly6C+ macrophages (iMo/Mac) are shown. Lower right panel shows the gating strategy and EGFP expression in MHC II^+ and MHC II^- Mac.

Racioppi et al. Supplementary Fig. 2

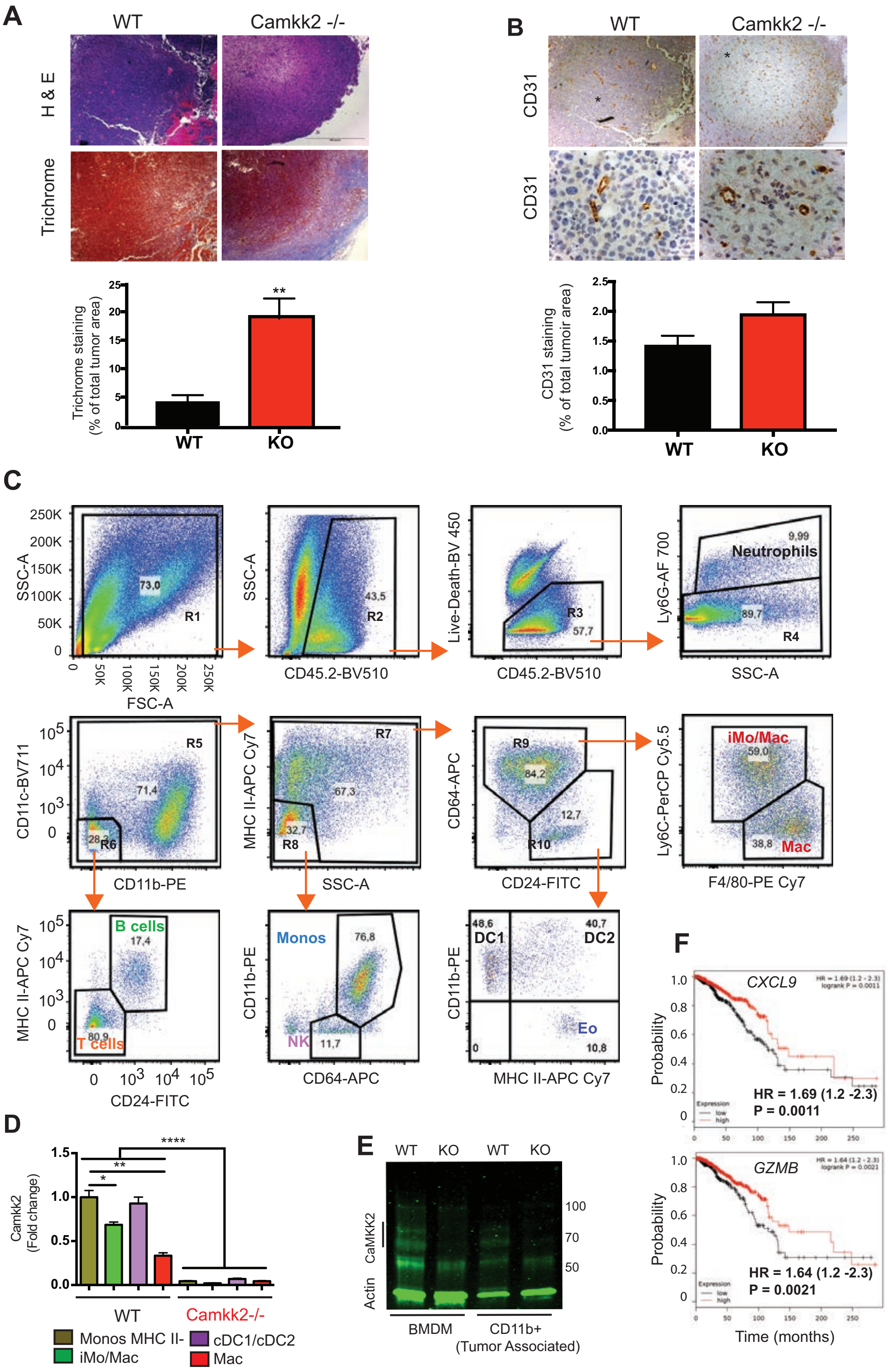

Supplementary Figure 2. Analysis of immune cell repertoire in E0771-cell derived tumors. Histological analysis of tumors removed from WT and *Camkk2*$^{-/-}$ mice. Representative images of (A) sequential tumor sections stained with hematoxylin and eosin (H & E) or Trichrome (upper and lower panels, respectively); (B) CD31 antibody staining at low and high-power optic field images (upper and lower panel, respectively). (A, B lower panels) Bar graphs report quantitation (Mean ± SEM) of Trichrome^{+} and CD31^{+} areas in stained sections (six fields for each section; 3 tumors for each group; N =18 data points/genotype). t-test was used to calculate p-values. **p<0.01. (C) E0771 tumors showing a comparable size (500-700 mm3) were removed from WT and *Camkk2*$^{-/-}$ mice and immune cell subsets were identified by flow cytometry. Dot plots show the gating strategy used to identify immune cell subsets. Stained tumor cell suspensions were then sorted by flow cytometry and myeloid cell subsets (Monos, iMo/Mac, cDC1/cDC2 and Mac) were directly collected in RNA lysis buffer for gene expression analysis. (D) *Camkk2* expression was evaluated by qPCR in myeloid cell subsets sorted from E0771 tumors removed from WT and *Camkk2*$^{-/-}$ mice (N=3 biological replicates for each group. Bar graph reports the mean ± SEM. t-test was used to calculate p-values. *p<0.05, **p<0.01, ****p<0.001, respectively. (E) CD11b^{+} cells were positively selected with magnetic beads from tumor single cell suspensions and cell lysates were used for protein analysis. Immunoblot shows CaMKK2 expression in bone marrow-derived macrophages (BMDM) and CD11b^{+} cells isolated from tumors grown in WT and *Camkk2*$^{-/-}$ mice. This experiment was replicated with similar results. (F) Increased expression of *CXCL9* and *GZMB* positively correlates with prognosis in patients with breast cancer. Kaplan-Meier plot of overall survival of breast cancer patients stratified by *CXCL9* and *GZMB* expression in the KM-Plotter Pan-Cancer RNA-seq database (http://kmplot.com/analysis). Log-rank test p-value is displayed.

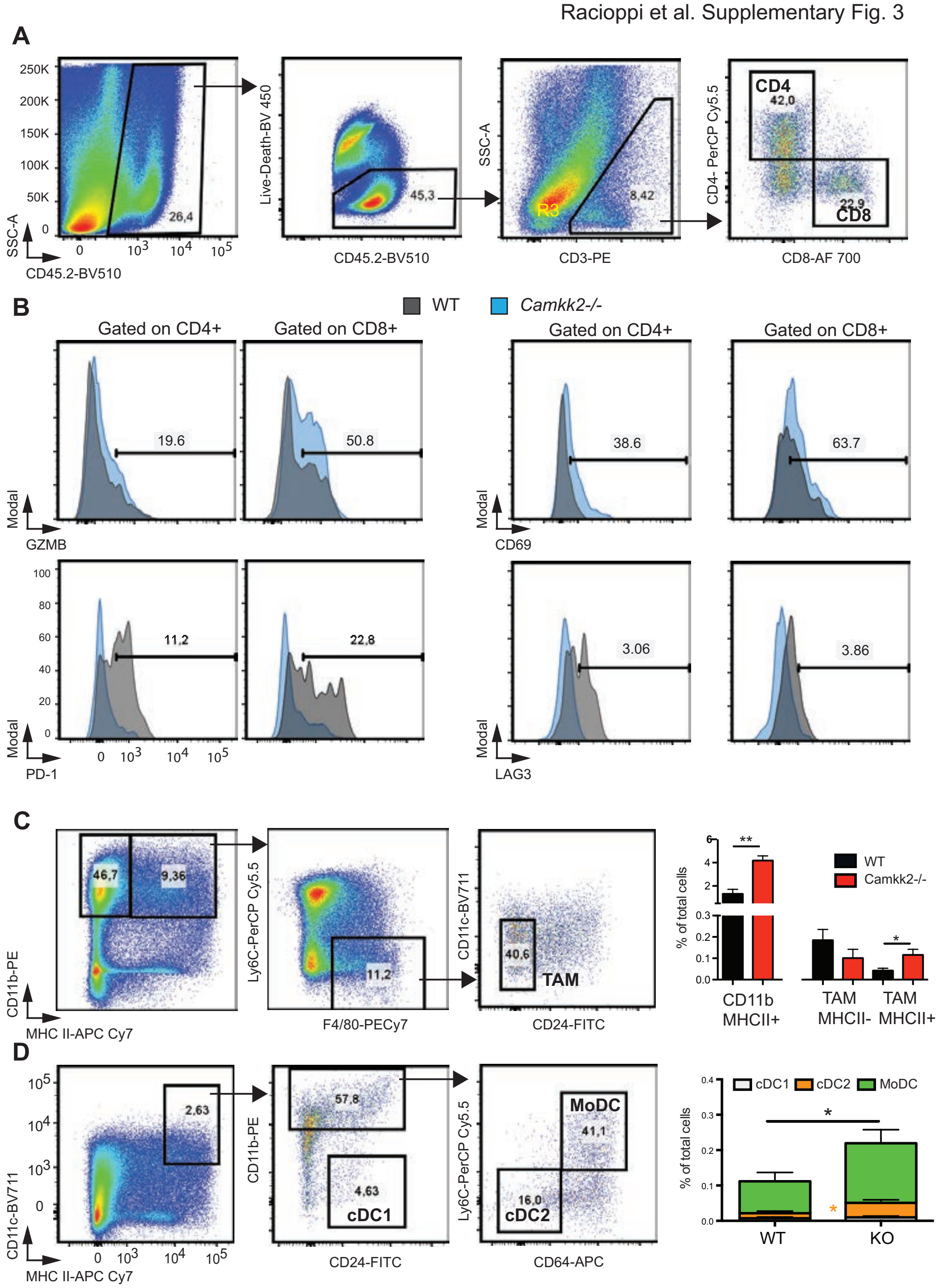

Supplementary Figure 3. Flow cytometry analysis of tumor-associated immune cells. (A) Gating strategy to identify $CD4^{+}$ and $CD8^{+}$ T cells. (B) Representative flow cytometry profiles showing GZMB, PD-1, CD69 and LAG-3 expression in T cell subsets. (C-D) Gating strategy to identify tumor-associated macrophages (TAM) and DC subsets (upper and lower left panels, respectively). Supplementary Figure 2C, R4 gate was used to sub-gate (TAM) and DC subsets. Bar graphs report mean ± SEM (as percentage of total cells) of myeloid cell subsets infiltrating E0771 tumors removed from WT and *Camkk2*$^{-/-}$ mice (N=8 and 4 tumors from WT and *Camkk2*$^{-/-}$ mice, respectively). t-test was used to calculate p-values (*p<0.05, **p<0.01, respectively).

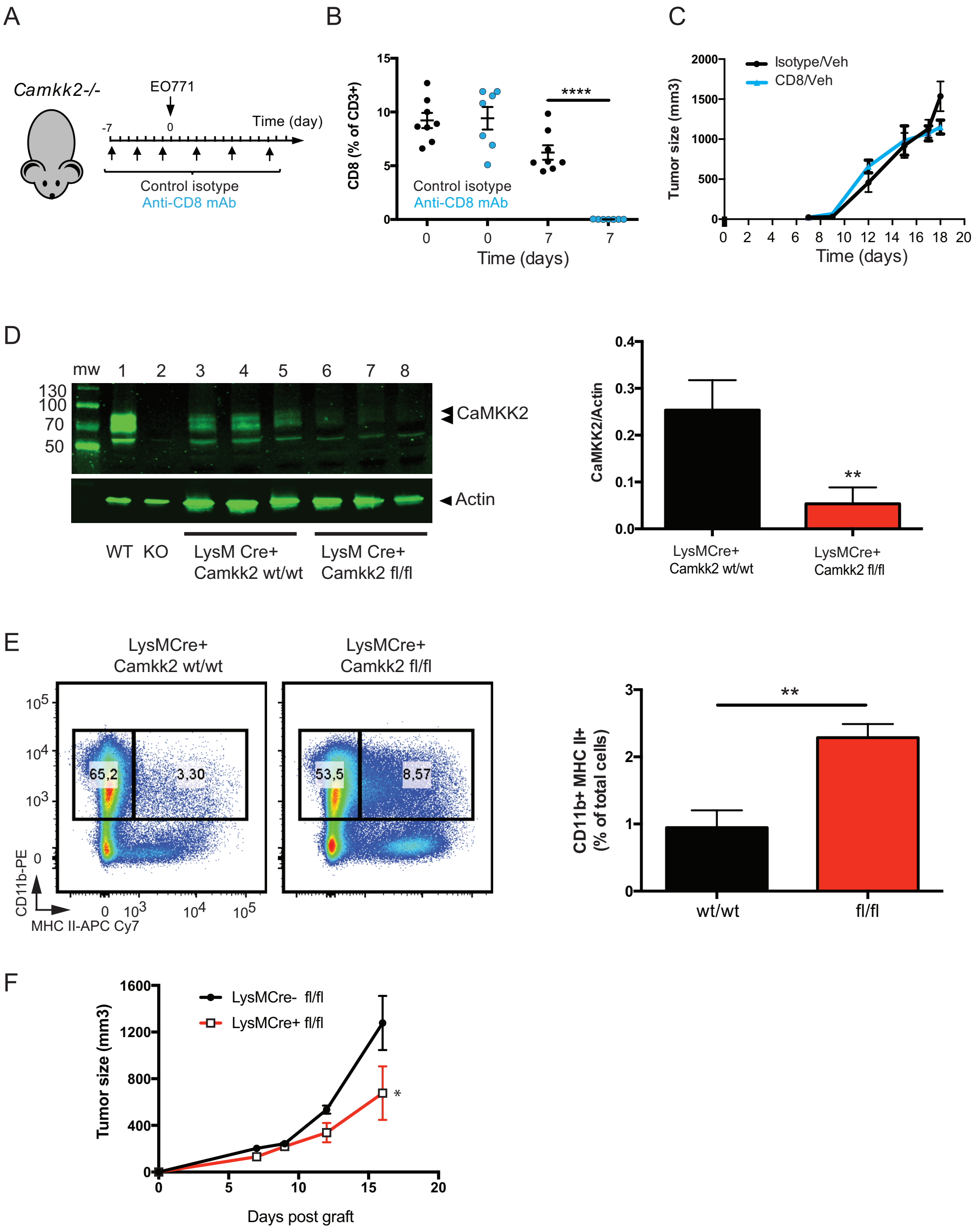

Supplementary Figure 4. Effects of CD8+ T cell depletion and *Camkk2* ablation in myeloid cells on E0771 mammary tumor growth. (A) Anti-CD8 or isotype control antibodies were inoculated intraperitoneally according to the regime outlined. Image in panel A was originally made by LR. (B) Graph reports the mean ± SEM of $CD8^+$ cells (as a percentage) in mouse peripheral blood collected before (day 0) and 7 days after E0771 cell engraftment. (C) Mammary tumor growth in $CD8^+$ cell depleted WT mice. (D) Left: Representative CaMKK2 immunoblot of protein

lysates from macrophages isolated by peritoneal lavage of LysMCre$^+$ *Camkk2*$^{wt/wt}$ and LysMCre$^+$ *Camkk2*$^{fl/fl}$ mice (N=3 each group). BMDM from WT and germline *Camkk2*$^{-/-}$ mice were used as positive and negative controls for CaMKK2 expression (lane 1 and 2, respectively). Right: Quantitation of immunoblot. (E) Dot plots show the gating strategy used to identify myeloid cell subsets in E0771 tumors removed from LysMCre$^+$ *Camkk2*$^{wt/wt}$ and LysMCre$^+$ *Camkk2*$^{fl/fl}$ mice (left). (Right) Bar graph reports Mean ± SEM of the percentage of CD11b+ MHC II+ cells (N=4 biological replicates for each group). t-test was used to calculate p-values (**p<0.01). (F) E0771 tumor growth in LysM-Cre$^-$ *Camkk2*$^{fl/fl}$ and LysM-Cre$^+$ *Camkk2*$^{fl/fl}$ mice (n= 5 mice each group). Two-way ANOVA test was used to calculate p-values. Asterisks refer to *p<0.05, **p<0.01 and ****p<0.001, respectively.

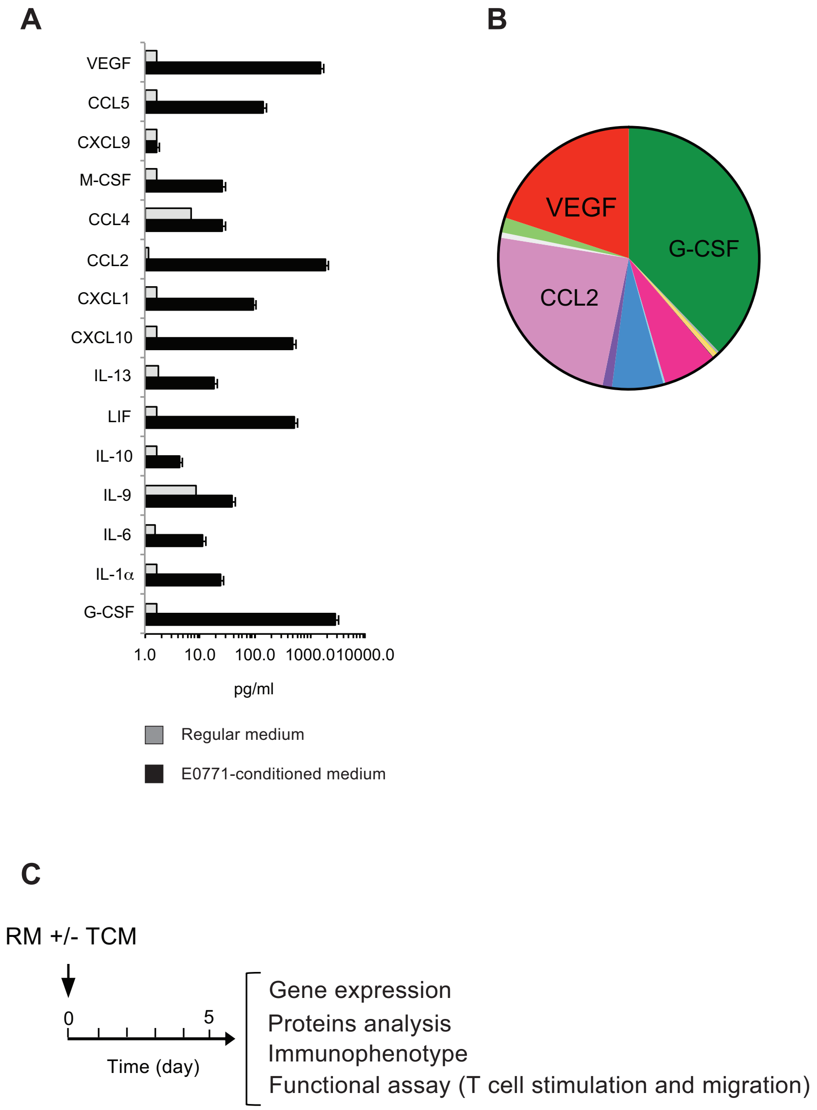

Supplementary Figure 5. Assessment of cytokines in E0771-conditioned medium. (A) Representative concentrations of cytokines detected in regular medium or in the culture supernatants from E0771 cells (N=3, technical replicates). (B) Graphical representation of the relative amounts of indicated cytokines in E0771-conditioned supernatants. (C) Experimental scheme used to determine the effects of E0771-conditioned medium on WT and *Camkk2*$^{-/-}$ bone marrow-derived macrophage development.

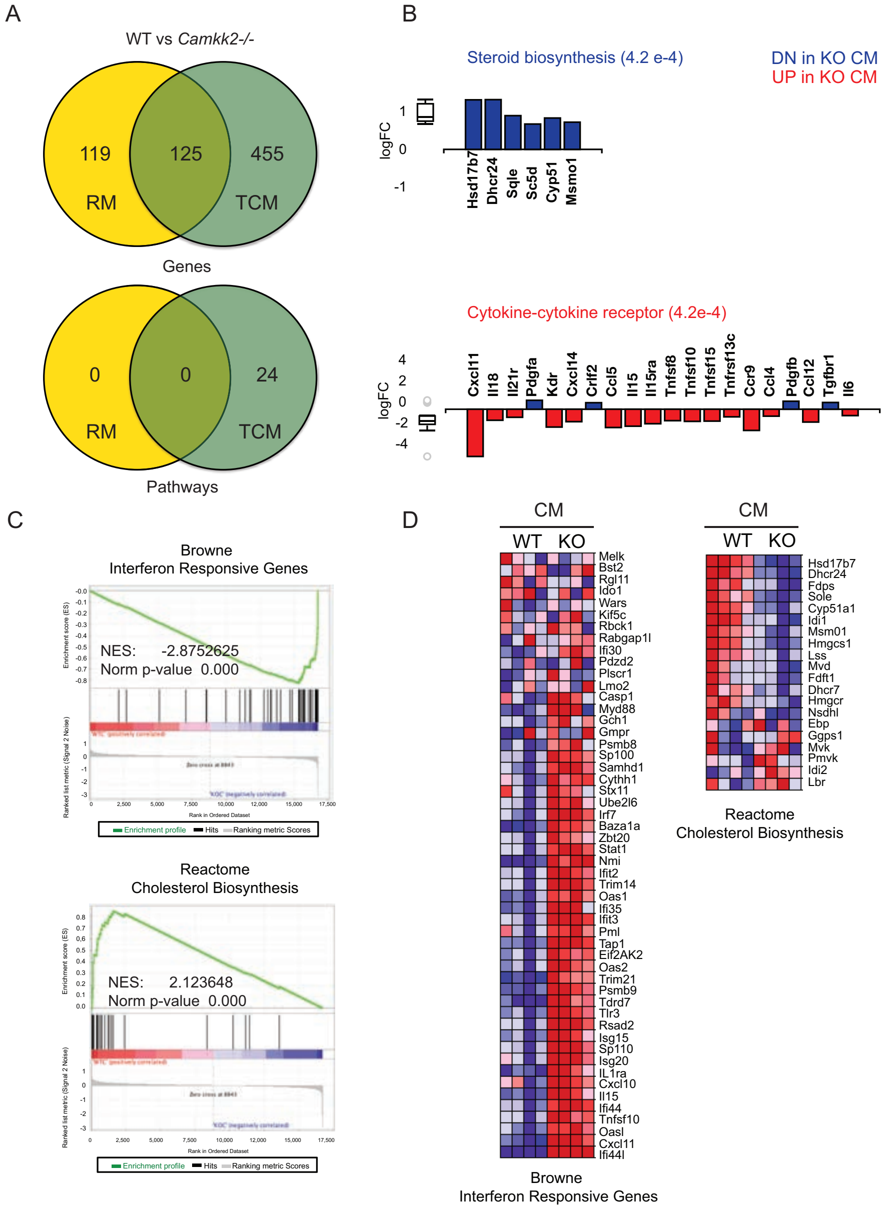

Supplementary Figure 6. *CaMKK2* regulates transcription in BMDM. (A) Venn diagrams showing the overlaps of DEGs and canonical pathways (top and bottom, respectively) in WT and *Camkk2*$^{-/-}$ BMDM generated in the presence of RM and TCM. (B) Expression of DEGs affiliated with steroid biosynthesis and cytokine-cytokines receptor signaling pathways down-regulated (DN) or up-regulated (UP) in *Camkk2*$^{-/-}$ TCM-BMDM (p-values are reported in parenthesis). (C) Gene Set Enrichment Analysis (GSEA) of microarray data shows that gene signatures for genes enriched in the interferon response pathway are up-regulated in *Camkk2*$^{-/-}$ TCM-BMDM compared to WT TCM-BMDM (top). In contrast, cholesterol biosynthesis genes are significantly down-regulated in *Camkk2*$^{-/-}$ TCM-BMDM compared to WT TCM-BMDM (bottom). (D) Heat-maps represent the expression of DEGs contained within gene sets of interferon response genes and cholesterol biosynthesis genes. The color key of heat-maps indicates row-wise scaled RPKM values (z-score).

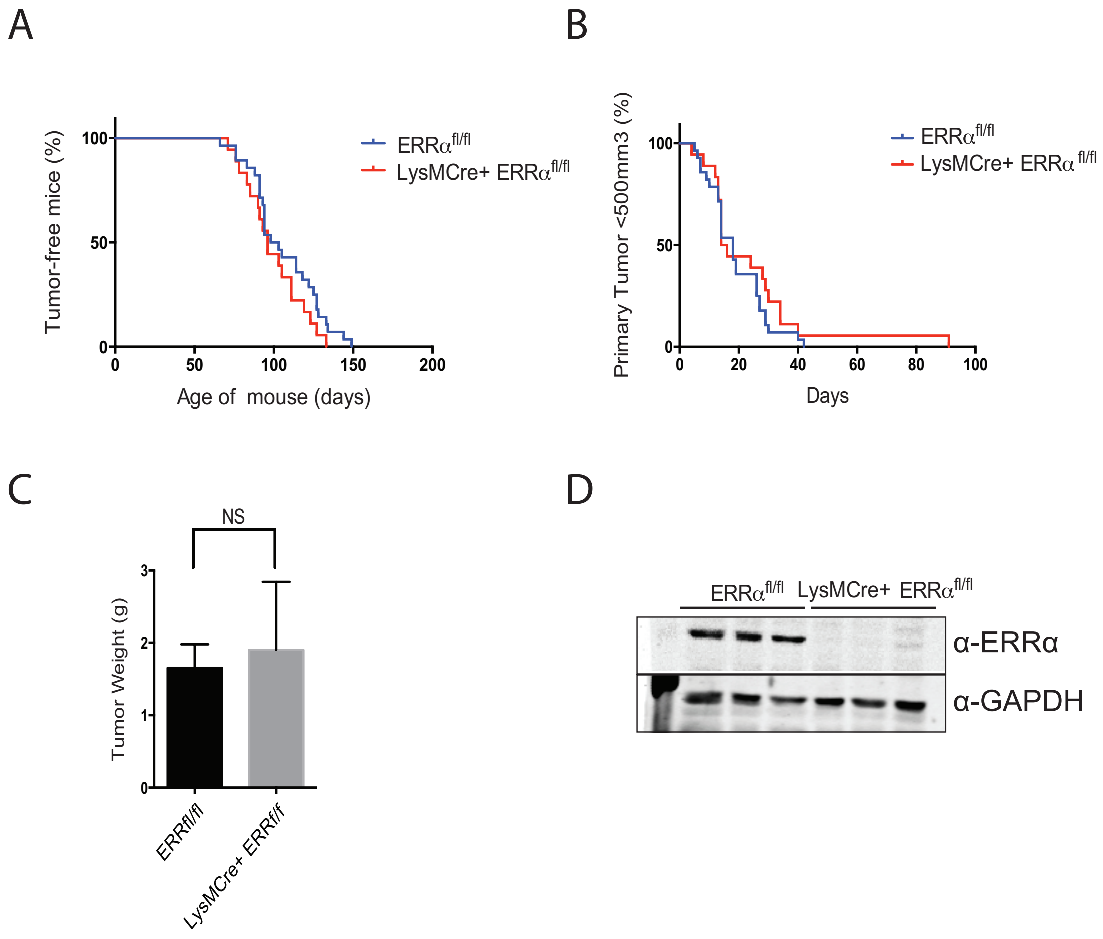

Supplementary Figure 7. Evaluation of potential downstream targets of CaMKK2 in macrophages. Myeloid cell-specific deletion of *ERRα* in the MMTV-PyMT mouse model of breast cancer. (A) Tumor latency, (B) tumor growth and (C) primary tumor weight at the time of harvest were measured in mice with myeloid cell-specific deletion of ERRα (LysMCre^{+} *ERRα*$^{fl/fl}$ n=30) and controls (*ERRα*$^{fl/fl}$ n=21). (D) Deletion of *ERRα* was confirmed by immunoblotting of bone marrow-derived macrophage (BMDM) isolated from *ERRα*$^{f/f}$ and LysMCre^{+} *ERRα*$^{fl/fl}$ mice.

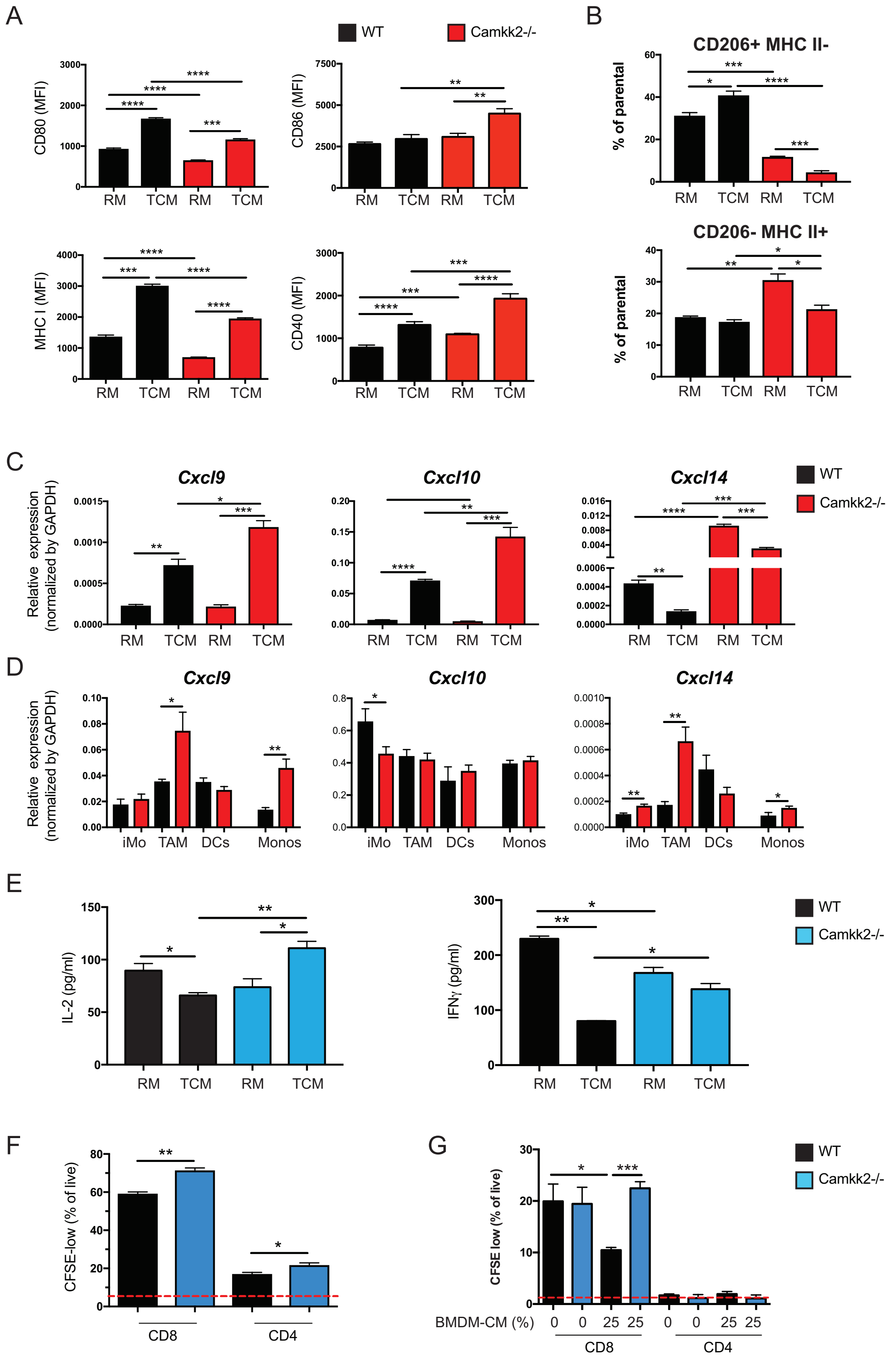
A
WT
Camkk2-/-
CD80 (MFI)
CD86 (MFI)
MHC I (MFI)
CD40 (MFI)
RM TCM RM TCM
B
CD206+ MHC II-
CD206- MHC II+
% of parental
C
Cxcl9
Cxcl10
Cxcl14
Relative expression (normalized by GAPDH)
D
iMo TAM DCs Monos
E
IL-2 (pg/ml)
IFNγ (pg/ml)
F
CFSE-low (% of live)
CD8
CD4
G
CFSE low (% of live)
BMDM-CM (%)
0 0 25 25 0 0 25 25

Supplementary Figure 8. Immune-stimulatory capability of WT and *Camkk2*$^{-/-}$ BMDM generated in the presence or absence of tumor-conditioned medium. (A) WT and *Camkk2*$^{-/-}$ BMDM were generated in the presence of RM or TCM. Bar graph report mean ± SEM (as mean fluorescent intensity, MFI) of CD80, CD86, MHC I and CD40 on CD11b^{+} F4/80^{+} gated cells (N=3 biological replicates for each group). (B) WT and *Camkk2*$^{-/-}$ BMDM were generated in the presence of RM. At day 4, 50% of cell culture media were removed and replaced with RM or TCM. At day 6, BMDM were harvested, stained with antibody mix and analyzed by flow cytometry. Bar graphs report percentage of CD206^{+}/MHC II^{-} and CD206^{-}/MHC II^{+} subsets (Mean ± SEM; N=3 biological replicates for each group). These experiments have been replicated at least 3 times with similar results. (C) The expression of *Cxcl9, Cxcl10* and *Cxcl14* RNAs were evaluated by qPCR on WT and *Camkk2*$^{-/-}$ BMDM generated in the presence of RM or TCM. Bar graphs report genes expression normalized by *Gapdh* (Mean ± SEM; N=3 biological replicates for each group). This experiment was replicated at least 3 times with similar results. (D) Bar graphs report expression of genes coding for chemokines in myeloid cell subsets sorted from E0771 tumors removed from WT and *Camkk2*$^{-/-}$ mice (Mean ± SEM; N=8 and 4 tumors removed from WT and *Camkk2*$^{-/-}$ mice, respectively). (E) BMDM were cultured with CFSE-labeled T cells isolated from wild type mice (1:100 BMDM/T cell ratio), in the presence of anti-CD3 antibody (10 ng ml^-1). Bar graphs report cytokines in cell supernatants following 24 h co-culture. (F) WT and *Camkk2*$^{-/-}$ BMDM generated in the presence of TCM (20% v/v) were cultured with purified CD4^{+} or CD8^{+} CFSE-labeled T cells isolated from wild type mice (1:100 BMDM/T cell ratio) in the presence of anti-CD3 antibody (10 ng ml^-1). After 72 hours, cells were harvested and T cells analyzed for CFSE staining. Bar graph reports the Mean ± SEM (as percentage) of CFSE-low CD8^{+} and CD4^{+} T cells. Red dotted line refers to the percentage of CFSE-low T cells cultured in the absence of BMDM. N=3 biological replicates for each genotype. The experiment was replicated with similar results. (G) Purified CD4^{+} or CD8^{+} CFSE-labeled T cells isolated from wild type mice (WT) and *Camkk2*$^{-/-}$ were stimulated with Dynabeads™ Mouse T-Activator CD3/CD28 (bead-to-cell ratio of 1:1) in the absence or presence of supernatants collected at day 5 from cultures of WT and *Camkk2*$^{-/-}$ TCM^{-}BMDM. Bar graphs report the Mean ± SEM (as percentage) of CFSE-low CD8^{+} and CD4^{+} T cells. Red dotted line refers to the percentage of CFSE-low T cells cultured in the absence of CD3/CD28 coated-beads. t-test was used to calculate p-values in all the experiments included in this figure. Asterisks refer to *p<0.05, **p<0.01, ***p<0.005 and ****p<0.001, respectively.

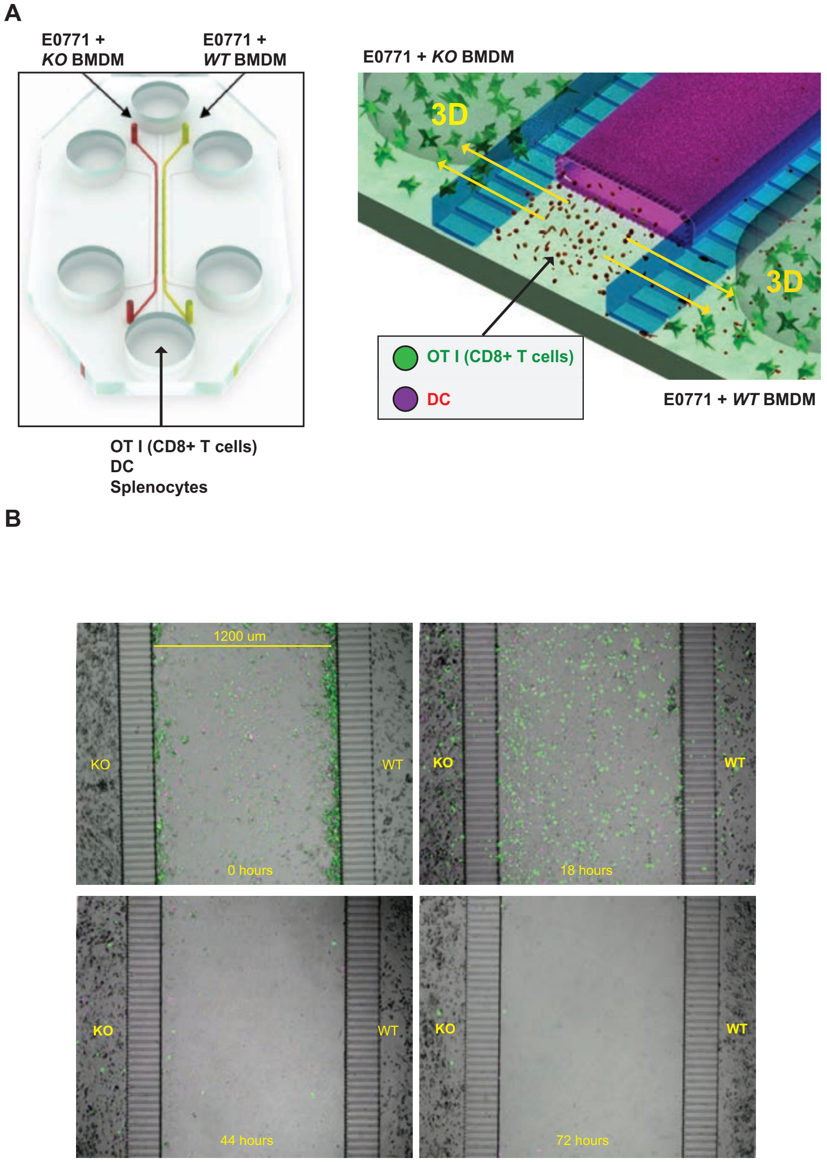

Supplementary Figure 9. Migration of OTI T cells toward E0771-tumor microenvironment chip. (A) General scheme of the device, composed of six reservoirs for cell loading and culture medium replacement and four chambers (or compartments) for cell culture. Tumor compartments are made by a collagen 3-D matrix (3D) including E0771 and BMDM (WT or *Camkk2 -/-*). Immune cell compartment includes GFP^{+} OTI cells and $DsRed^{+}$ bone marrow-derived DC (BMDC). (B) Detailed view of the device. Far left and far right chambers are dedicated to E0771/*Camkk2*$^{-/-}$ BMDM and E0771/WT BMDM tumor compartments, respectively. Immune cells (OTI and BMDC), initially loaded in the middle chamber (immune chamber), sense the attractant signals and start to migrate towards tumor compartments through the micro-channels connecting the immune chamber with two tumor microenvironment chambers. (C) Examples of the typical responses performed by OTI T cells toward tumor compartments containing CaMKK2 WT and KO BMDMs. Panel A was originally made by LB.

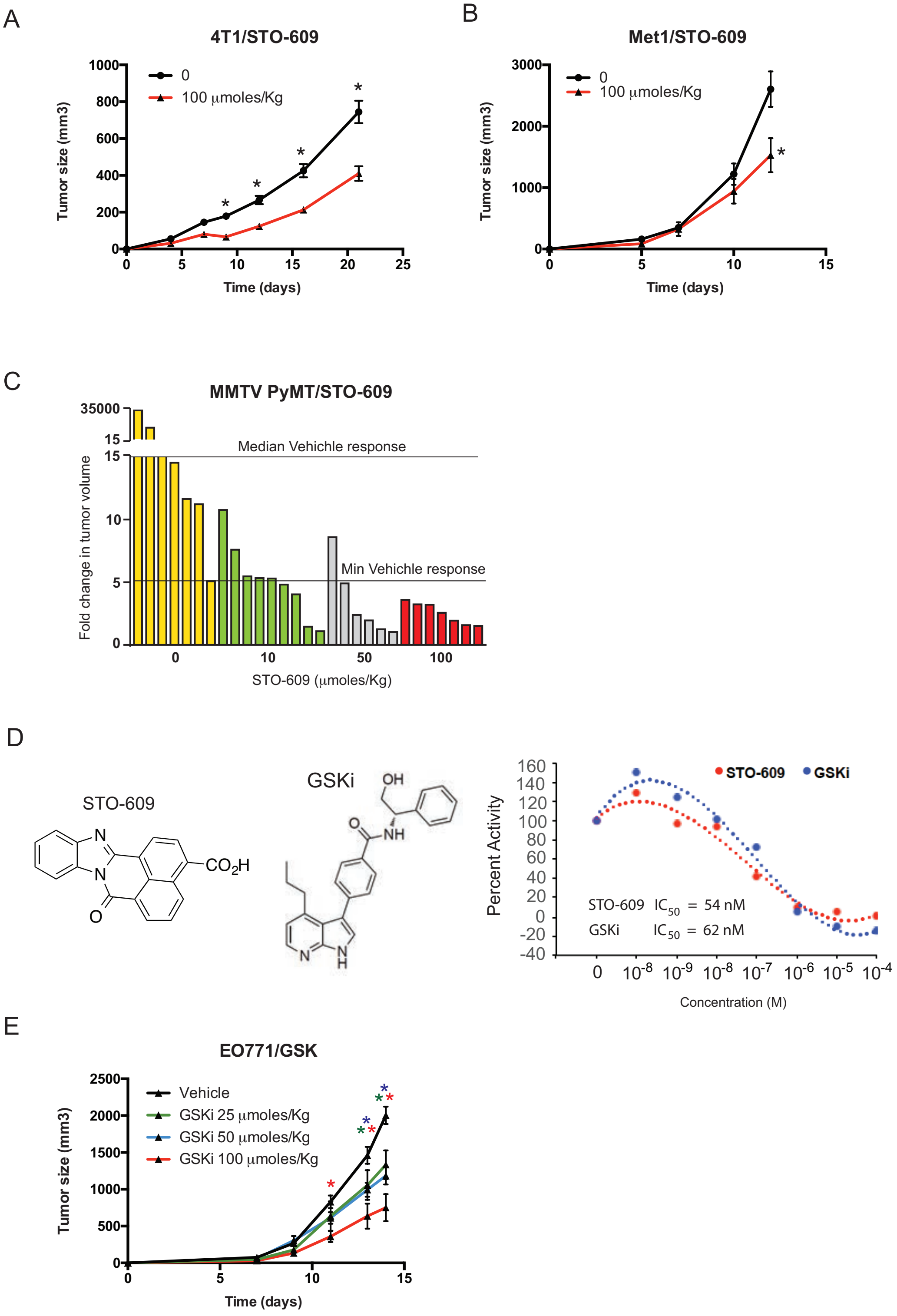
A
4T1/STO-609
0
100 µmoles/Kg
Tumor size (mm3)
Time (days)
B
Met1/STO-609
0
100 µmoles/Kg
Tumor size (mm3)
Time (days)
C
MMTV PyMT/STO-609
Median Vehichle response
Min Vehichle response
Fold change in tumor volume
STO-609 (µmoles/Kg)
D
STO-609
GSKi
STO-609
GSKi
Percent Activity
STO-609 IC50 = 54 nM
GSKi IC50 = 62 nM
Concentration (M)
E
EO771/GSK
Vehicle
GSKi 25 µmoles/Kg
GSKi 50 µmoles/Kg
GSKi 100 µmoles/Kg
Tumor size (mm3)
Time (days)

Supplementary Figure 10. Small molecule inhibitors of CaMKK2 inhibit breast cancer tumor growth. (A-B) Mammary tumor cells (4 x 10^5/mouse for 4T1 and 5 x 10^5/mouse for Met1) were orthotopically grafted into syngeneic WT C57BL/6 mice. Mice were treated 3 times/week with vehicle or STO-609 (IP, 100 μmoles/kg body weight), and subsequently tumor volume measured (mean ± SEM; N=5 in each group). The growth of 4T1 and Met1 mammary tumors were inhibited by STO-609 treatment. (C) Waterfall-plot reports fold change in tumor volumes of mice bearing MMTV-PyMT mammary tumor treated daily with vehicle or indicated dose of STO-609, a CaMKK2 inhibitor. (D) Two-steps *in vitro* CaMKK2 kinase assay. (Left) Structures of the CaMKK2 inhibitors STO-609 and GSKi. (Right) Purified recombinant GST-CaMKK2 (4 nM) was incubated with MBP-AMPK$\alpha2^{1-312}$ (200 nM) and SAMS peptide (20 μM) in the presence of [γ^{32}P]-ATP ± CaMKK2 inhibitors or ± Ca^{2+}/CaM as described by Green et al.[1]. Incorporation of labeled ^{32}P on SAMS peptide (an AMPK substrate) was measured by scintillation counting as a readout of CaMKK2 and/or AMPKα2 phosphorylation. Reaction controls for no CaMKK2, no AMPK or no SAMS peptide were included to determine background and signal specificity. Assay results showing equal efficacy of two structurally unique CaMKK2 inhibitors. STO-609 IC_{50} = 54 nM; GSKi IC_{50} = 62 nM. (E) E0771 mammary tumor growth is inhibited by the GSKi CaMKK2 inhibitor in a dose-dependent manner.

Racioppi et al. Supplementary Fig. 11

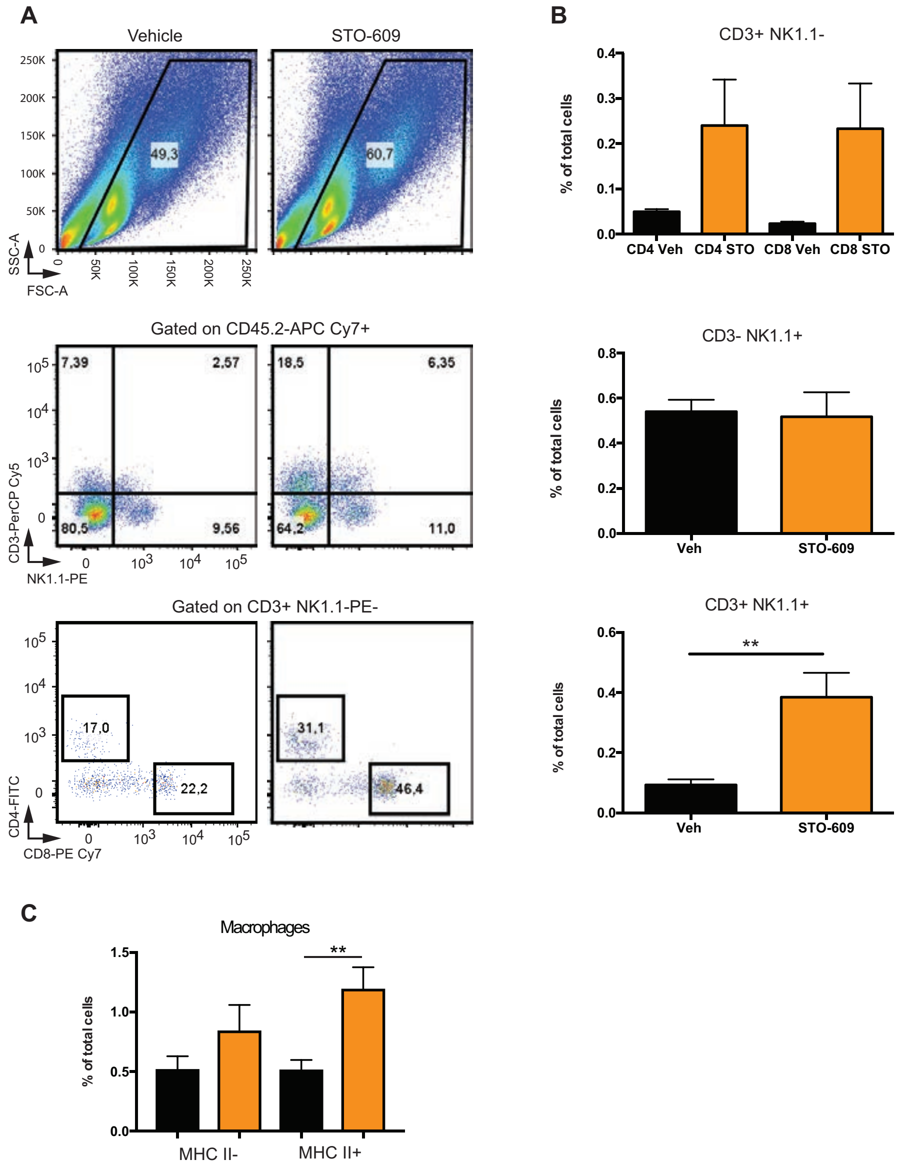

Supplementary Figure 11. Effects of STO-609 treatment on E0771 tumor microenvironment. E0771 mammary tumors were removed from STO-609 or vehicle-treated mice, dissociated as described previously and single cell suspensions were stained for myeloid or T cell markers. (A) Gating strategy used to identify lymphocyte subsets infiltrating E0771 tumors. (B) Graphs showing mean ± SEM (as percentage of total cells) of CD3$^+$ NK1.1$^-$ CD4$^+$ and CD8$^+$ T cells (top), CD3$^-$ NK1.1$^+$ NK cells (middle) and CD3$^+$ NK1.1$^+$ NK-T cells (bottom) infiltrating tumors from mice treated with STO-609 or vehicle (N=5 and 4 mice for Vehicle and STO-609 treated groups, respectively). (C) Graph reports mean ± SEM (as percentage of total cells) of tumor-associated macrophages (gating strategy shown in Supplementary Figures 2C and 3C). t-test was used to calculate p-values. Asterisks refer to **$p<0.01$.

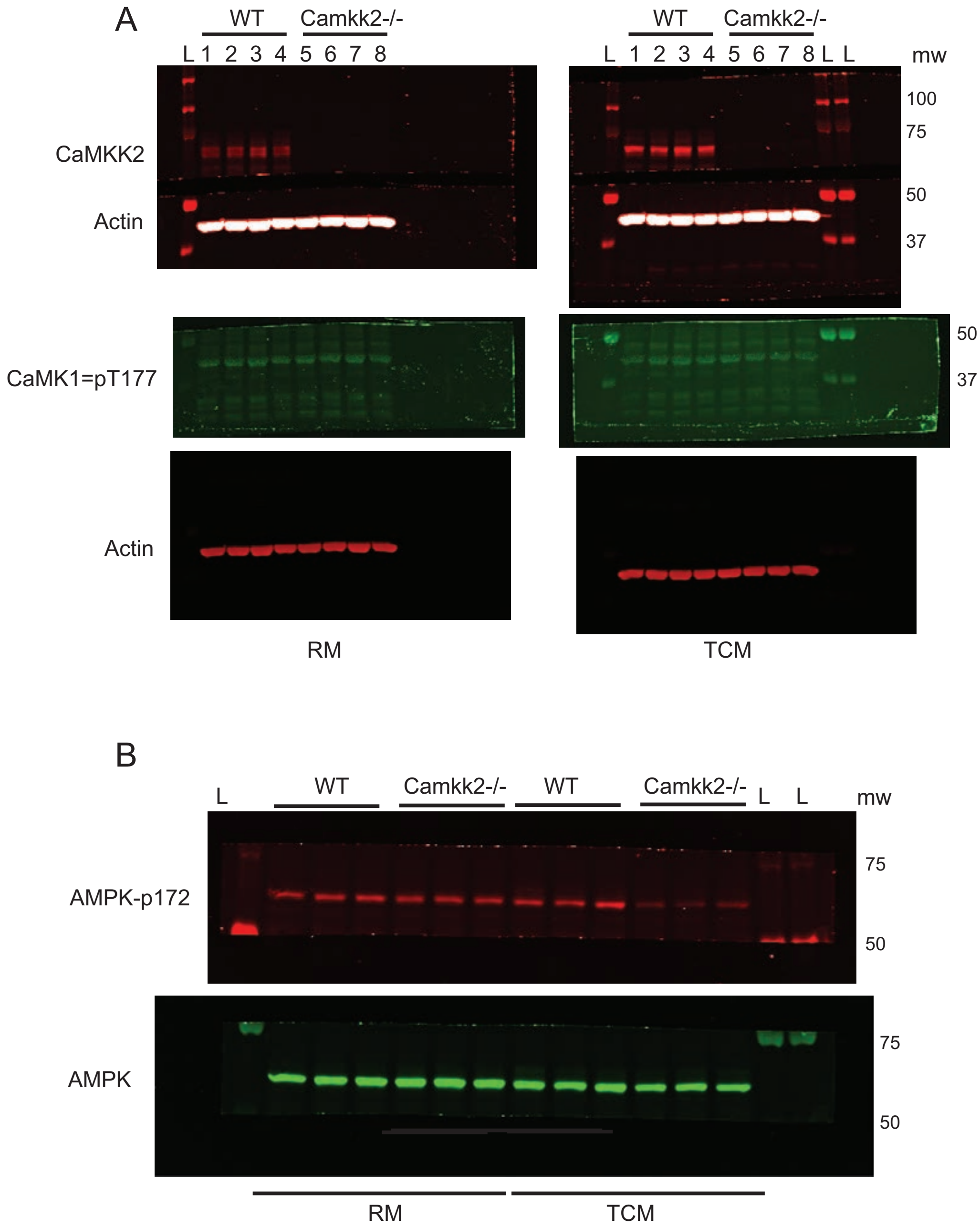

Supplementary Figure 12. Uncropped and unmanipulated immunoblots displayed in Figure 5.
