# Supplementary material for: CaMKK2 in myeloid cells is a key regulator of the immune-suppressive microenvironment in breast cancer
Source: Nat Commun. 2019 Jun 4;10:2450. doi: 10.1038/s41467-019-10424-5 (PMC6547743; doi:10.1038/s41467-019-10424-5)
Supplement: Supplementary file 4 — Description of Additional Supplementary Files [file 41467_2019_10424_MOESM4_ESM.docx]

**Title: Supplementary Data 1**
**Description:** List of the differentially expressed genes (DEGs) in WT and Camkk2- /- macrophages. BMDM were generated in the presence of regular medium or tumor-conditioned medium (RM or TCM) and microarray analysis was then performed. Differentially expressed genes (DEGs) were identified based on LogFc (< -0.5 or > + 0.5) and adjusted p-value (< 0.05).

**Title: Supplementary Data 2**
**Description:** List of significantly over-represented pathways in WT and Camkk2-/- macrophages. BMDM were generated in the presence of regular medium or tumor-conditioned medium (RM or TCM) and microarray analysis was then performed. Significantly over-represented pathways were identified in DEGs.
